# Polymorphisms in human telomerase reverse transcriptase (hTERT) gene, gene- gene and gene-smoking interaction with susceptibility to gastric cancer in Chinese Han population

**DOI:** 10.18632/oncotarget.15664

**Published:** 2017-02-24

**Authors:** Jian Zhang, Hui Ju, Jun- Ru Gao, Xue-Long Jiao, Yun Lu

**Affiliations:** ^1^ Department of General Surgery, The Affiliated Hospital of Qingdao University, Qingdao 266003, Shandong Province, People's Republic of China

**Keywords:** gastric cancer, TERT gene, single nucleotide polymorphisms, interaction, haplotype

## Abstract

**Aims:**

To investigate the association of telomerase reverse transcriptase (*TERT*) gene polymorphisms and additional gene-gene and gene- environment interaction with gastric cancer (GC) risk.

**Results:**

GC risk was significantly higher in carriers of G allele of rs2736100 than those with TT genotype (TG+ GG versus TT), adjusted OR (95%CI) =1.68 (1.26-2.17), and higher in carriers of G allele of rs2853669 than those with AA genotype (AG+ GG versus AA), adjusted OR (95%CI) = 1.72 (1.19-2.33). We also found that interaction between rs2736100 and smoking was associated with higher GC risk. Smokers with TG or GG of rs2736100 genotype have elevated GC risk, compared to never- smokers with TT of rs2736100 genotype, OR (95%CI) = 3.12 (1.82 -4.61). Pairwise linkage equilibrium (LD) analysis between SNPs was measured and the D’ value between rs2736100 and rs2736109 was more than 0.8. A haplotype containing the rs2736100- G and rs2736109- A alleles was associated with a statistically increased GC risk (OR= 2.66, 95%CI= 1.28 – 4.12, *p*<0.0001).

**Materials and Methods:**

A total of 1088 participants (686 males, 402 females) were selected, including 360 GC patients and 728 normal participants. Logistic regression was performed to investigate association between single nucleotide polymorphisms (SNPs) within *TERT* gene and GC susceptibility. Generalized multifactor dimensionality reduction (GMDR) model was used to screen gene- gene and gene- environment interaction combinations.

**Conclusions:**

We found that G allele of rs2736100 and G allele of rs2853669 in *TERT* gene, interaction between rs2736100 and smoking, and haplotype containing the rs2736100- G and rs2736109- A alleles were all associated with increased GC risk.

## INTRODUCTION

Gastric cancer (GC) is one of the most common types of tumor. In the world, almost one million new cases of GC are estimated to have been diagnosed in 2012 (952,000 cases, 6.8% of the total GC cases), making it the fifth most common malignancy [1]. More than 70% of cases (677,000 cases) occur in developing countries (456,000 in men, 221,000 in women), and half of the world total occurs in Eastern Asia (mainly in China) [1]. Some studies have reported that risk factors, such as consumption of alcohol, lack of activity, obesity and high sodium intake may play critical roles in susceptibility to GC [2]. However, not all individuals exposed to these factors have developed GC, suggesting that genetic factors may play an important role in GC development. In recent years, some genes involved in GC risk have been reported, including microRNAs -146a, interleukin-17A (IL-17A), low molecular weight protein 2 (LMP2), LMP7, telomerase reverse transcriptase (*TERT*) and so on [3–6].

TERT, as the reverse transcriptase component of telomerase, plays critical roles in maintenance of telomeres, chromosome stability and preventing malignancy [7]. Apart from the telomere elongation, many biological functions of *TERT* have been shown to be associated with tumorigenesis and tumor progression. In humans, telomeres are composed of tandem nucleotide repeats of the (TTAGGG)_n_ sequence, that are bound by a multi- protein complex also known as “shelterin” or the telosome which has basic roles in the arrangement of telomerase activity [8, 9]. Several common functional single nucleotide polymorphisms (SNPs) in the promoter region of *TERT* gene were reported in previous studies [10–13], including rs2736109, rs2735940. However, till now, just one study involved in relation between SNP within *TERT* gene and GC risk was conducted. It is generally considered that the development of GC is the result of a combination of environmental and genetic risk factors as well as gene- gene and gene- environment interactions. Cigarette smoking is a well-known risk factor leading to cancer. Cigarette smoking compromises the immune system by suppressing the activation of immune cells and increasing susceptibility to infections. Several studies have confirmed the association between smoking and GC risk [14, 15]. Several studies also reported association of gene- smoking interactions with GC risk, however no study focused on impact of *TERT* gene - smoking interactions on GC risk. So in current study, we aimed to investigate the association of several SNPs in *TERT* gene and additional gene- gene and gene- smoking interaction with GC risk in a Chinese population.

## RESULTS

Participants characteristics stratified by cases and controls are shown in Table 1. The means of age and distribution of males, smokers, alcohol drinkers were not significantly different between cases and controls. The mean of BMI was higher in controls than that in cases. The rate of positive *Helicobacter pylori*, non-cardia location and tumor size ≤5 cm were 49.2%, 62.5% and 73.3% perspective.

**Table 1:** General characteristics of 1088 study participants in case and control group

Variables	Case group (n=360)	Normal group (n=728)	*p-*values
Age (year) (Means± SD)	60.8±14.7	61.9±15.1	0.254
Males, N (%)	230 (63.9)	456 (62.6)	0.687
Smokers, N (%)	121 (33.6)	218 (29.9)	0.219
Alcohol drinkers, N (%)	139 (38.6)	246 (33.8)	0.118
BMI (kg/m^2^) (Means± SD)	23.1±9.0	24.6±9.2	0.011
Family history of GC N (%)	43 (11.9)		
*Helicobacter pylori*			
Positive	177 (49.2)		
Negative	183 (50.8)		
Tumor location			
Non-Cardia	225 (62.5)		
Cardia	135 (37.5)		
Tumor size			
≤5 cm	264 (73.3)		
>5 cm	96 (26.7)		

BMI: body mass index; GC: gastric cancer; SD: standard deviation; N: number

All genotypes were distributed according to Hardy–Weinberg equilibrium in controls (all *p* values were more than 0.05). The frequencies for G allele of rs2736100 and rs2853669 in *TERT* gene were significantly higher in GC cases than that in control group (28.8% *vs*19.5%, 31.1% *vs*20.0%). Logistic regression analysis showed that GC risk was significantly higher in carriers of G allele of rs2736100 than those with TT genotype (TG+ GG versus TT), adjusted OR (95%CI) =1.68 (1.26-2.17), and higher in carriers of G allele of rs2853669 than those with AA genotype (AG+ GG versus AA), adjusted OR (95%CI) = 1.72 (1.19-2.33). However, we found that rs2736109 and rs2735940 were not associated with GC risk after adjustment for covariates (Table 2).

**Table 2:** Genotype and allele frequencies of 4 SNPs between case and control group

SNP	Genotypes and Alleles	Frequencies N (%)		OR(95%CI)*	HWE test for controls
**Control (n=728)**	**Case (n=360)**
rs2736100					
	Codominant				
	TT	476 (65.4)	186 (51.7)	1.00	0.310
	TG	220 (30.2)	141 (39.2)	1.47 (1.22-1.85)	
	GG	32 (4.4)	33 (9.1)	2.12 (1.43-2.88)	
	Dominant				
	TT	476 (65.4)	186 (51.7)		
	TG+GG	252 (34.6)	174 (48.3)	1.68 (1.26-2.17)	
	Allele, G (%)	284 (19.5)	207 (28.8)		
rs2853669					
	Codominant				
	AA	473(65.0)	180(50.0)	1.00	0.109
	AG	219(30.1)	136(37.8)	1.36(1.08-1.72)	
	GG	36(4.9)	44(12.2)	2.38(1.59-3.17)	
	Dominant				
	AA	473(65.0)	180(50.0)		
	AG+GG	255(35.0)	180(50.0)	1.72(1.19-2.33)	
	Allele, G (%)	291(20.0)	224(31.1)		
rs2736109					
	Codominant				
	GG	417(57.3)	185(51.4)	1.00	0.104
	GA	257(35.3)	139(38.6)	1.25 (0.82-1.81)	
	AA	54 (7.4)	36(10.0)	1.49 (0.87-2.15)	
	Dominant				
	GG	417(57.3)	185(51.4)		
	GA+AA	311(42.7)	175(48.6)	1.31(0.84-1.90)	
	Allele, A (%)	365(25.1)	211(29.3)		
rs2735940					
	Codominant				
	TT	425(58.4)	189 (52.5)	1.00	0.934
	TC	263(36.1)	142 (39.4)	1.29(0.83-1.86)	
	CC	40(5.5)	29 (8.1)	1.54(0.80-2.31)	
	Dominant				
	TT	425(58.4)	189 (52.5)		
	TC+CC	303(41.6)	171(47.5)	1.35(0.82-1.93)	
	Allele, C (%)	343(23.6)	200(27.8)		

^*^Adjusted for gender, age, smoking and alcohol status, BMI and WC. HWE: Hardy-Weinberg equilibrium

We also investigated the impact of the interaction among 4 SNPs within *TERT* gene and gene- environment interaction on GC risk by using GMDR model. Table 3 summarizes the results obtained from GMDR analysis for gene- gene and gene- environment interactions; however we did not find any significant two-locus model in gene- gene interaction analysis. We found a significant two-locus model (p=0.0100) involving rs2736100 and smoking, and the cross-validation consistency of this model was 10/ 10. Testing accuracy was 60.72%. But we did not find any significant gene- environment interaction between SNP within *TERT* gene and BMI or alcohol drinking. We also conducted analysis on interaction between rs2736100 and smoking by using logistic regression. We found that smokers with TG or GG of rs2736100 genotype have the highest GC risk, compared to never- smokers with TT of rs2736100 genotype, OR (95%CI) = 3.12 (1.82 -4.61), after adjustment for covariates (Table 4).

**Table 3:** GMDR analysis on the best gene–gene and gene- smoking interaction models

Locus no.	Best combination	Cross-validationconsistency	Testingaccuracy	*p-values*
Gene- gene interactions*				
2	rs2736100 rs2853669	9/10	0.5590	0.0547
3	rs2736100 rs2853669 rs2735940	8/10	0.5399	0.1719
4	rs2736100 rs2853669 rs2735940 rs2736109	7/10	0.4958	0.3770
Gene- smoking interactions ^*^				
2	rs2736100 Smoking	10/10	0.6072	0.0100
3	rs2736100 rs2853669 Smoking	9/10	0.5399	0.1719
4	rs2736100 rs2853669 rs2735940 Smoking	8/10	0.4958	0.3770
5	rs2736100 rs2853669 rs2735940 rs2736109 Smoking	7/10	0.4958	0.4258
Gene- BMI interactions ^#^				
2	rs2853669 BMI	9/10	0.5399	0.0547
3	rs2853669 rs2736100 BMI	8/10	0.5399	0.0547
4	rs2853669 rs2736100 rs2735940 BMI	7/10	0.4958	0.3770
5	rs2853669 rs2736100 rs2735940 rs2736109 BMI	5/10	0.4958	0.9893
Gene- alcohol drinking interactions ^##^				
2	rs2853669 alcohol drinking	8/10	0.5399	0.1719
3	rs2853669 rs2736100 alcohol drinking	7/10	0.4958	0.3770
4	rs2853669 rs2736100 rs2735940 alcohol drinking	6/10	0.4958	0.6230
5	rs2853669 rs2736100 rs2735940 rs2736109 alcohol drinking	6/10	0.4958	0.6230

GMDR: Generalized multifactor dimensionality reduction

*Adjusted for gender, age, smoking, drinking and BMI for gene- gene interaction analysis

**Adjusted for gender, age, drinking and BMI for gene- smoking interaction analysis

^#^ Adjusted for gender, age, smoking and drinking for gene- BMI interaction analysis

^##^ Adjusted for gender, age, smoking and BMI for gene- alcohol drinking interaction analysis

**Table 4:** Interaction analysis for rs2736100 and smoking by using logistic regression

rs2736100	Smoking	OR (95% CI)*	*P-values*
TT	Never	1.00	-
TG or GG	Never	1.48 (1.14 -1.92)	0.001
TT	Current	1.38 (1.05-1.79)	0.032
TG or GG	Current	3.12 (1.82 -4.61)	<0.001

^*^Adjusted for gender, age, drinking and BMI

Pairwise LD analysis among SNPs was measured and all D’ values are shown in Table 5. Just the D’ value between rs2736100 and rs2736109 was more than 0.8. The most common haplotype was rs2736100- T and rs2736109- G haplotype, the frequency of which was 0.4228 and 0.4910 in case and control group. Haplotype containing the rs2736100- G and rs2736109- A alleles was associated with a statistically increased GC risk (OR= 2.66, 95%CI= 1.28 – 4.12, *P*<0.0001) (Table 6).

**Table 5:** The D’ values among 4 SNPs within TERT gene for the linkage disequilibrium test

SNPs	D’ values		
**rs2853669**	**rs2735940**	**rs2736109**
rs2736100	0.148	0.423	**0.871**
rs2853669	-	0.312	0.028
rs2735940	-	-	0.354

**Table 6:** Haplotype analysis on association between TERT gene and GC risk

Haplotypes	rs2736100	rs2736109	Frequencies		OR(95%CI)	*p*-values*
**Case group**	**Control group**
H1	**T**	**G**	0.4228	0.4910	1.00	--
H2	**T**	**A**	0.2709	0.2690	1.21(0.85 - 1.67)	0.421
H3	**G**	**G**	0.1995	0.1860	1.16 (0.69 - 1.85)	0.708
H4	**G**	**A**	0.1068	0.0540	2.66 (1.28 – 4.12)	0.00

^*^Adjusted for gender, age, smoking, drinking and BMI

## DISCUSSION

In this study, we found that both G allele of rs2736100 and G allele of rs2853669 in *TERT* gene were associated with increased GC risk, but we did not find any relation of rs2736109 and rs2735940 within *TERT* gene with GC risk. TERT rs2853669 ATG polymorphism is located −245 bp upstream from the “A>G” transcription initiating site of the *TERT* promoter region, residing within a particular binding motif “GGAA/T” for E-twenty six-2 (Ets-2) transcription factor [14]. A transition from A>G of *TERT* rs2853669 A>G polymorphism results in the disruption of a pre-existing non-canonical Ets-2 core binding site motif adjacent to an E-box, which affects the binding ability of Ets-2 and therefore the transcriptional efficiency and expression level of the *TERT* gene [14]. Some population based studies have reported the associations of this gene with some types of cancer. A recent study reported that minor allele of rs2736098 and rs2736100 in *TERT* gene and interaction between the two SNP were associated with increased risk of non-small cell lung cancer (NSCLC) [15]. Yoo et al [16] showed that GG genotype of *TERT* rs2853669 A>G was significantly associated with increased lung cancer (LC) risk under a recessive model in the Korean population. Zhong et al [17] conducted a study for Chinese population and suggested that GG genotype of *TERT* rs2853669 A>G was also significantly associated with increased LC risk, especially with non-small lung cancer. Some other studies also reported the association of this gene with acute myeloid leukemia [18], prostate cancer [19] and breast cancer [20]. Although these studies have focused on the associations between TERT SNP and some type cancer diseases, however, few study focused on the relationship between TERT SNP and GC risk. In addition, in these previous studies, few studies reported the impact of gene- gene interaction among several SNPs and gene—environment risk factors interaction on GC risk. Till now, just one study conducted by Bayram et al [21] focused on the association between *TERT* gene and GC risk. They found for the first time that *TERT* rs2736109 G>A, rs2735940 T>C and rs2736100 T>G polymorphisms were associated with the risk of GC susceptibility, but they did not find association between rs2853669 and GC risk. Regarding *TERT* rs2736100 polymorphism, the association between this SNP and cancer risks was also reported in previous case- control and meta- analysis studies. Lan et al [22] suggested that G allele and/or GG genotype of *TERT* 2736100 T>G polymorphism was significantly associated with an increased risk of LC susceptibility. The findings obtained by Bayram et al [21] also support previous data showing that there was an association between the G allele and/or GG genotype of *TERT* rs2736109 G>A polymorphism and increased risk for human cancers. However, Jannuzzi et al [23] reported that there was no correlation between *TERT* 2736100 polymorphism and susceptibility to hepatocellular carcinoma. Our study is the second study focused on the association between *TERT* gene and GC risk, and we concluded similar results with Bayram et al [21] on relationship between rs2736100 polymorphism and GC.

Several environmental factors associated with GC were reported previously, and in which cigarette smoking has been suggested to play a crucial role in increasing the risk of GC. Previous epidemiologic studies indicated that cigarette smoking was an independent risk factor for gastric cancer development [14, 15]. It has been increasingly accepted that the etiology of most common tumors involves not only genetic, but environmental causes, or interactions between the two. So it was meaningful to investigate the impact of gene- environment interaction between *TERT* gene and cigarette smoking on GC risk. In this study, we found a significant gene– smoking interaction between rs2736100 and smoking on GC, and smokers with TG or GG of rs2736100 genotype have the highest GC risk, compared to never- smokers with TT of rs2736100 genotype. This is the first study focused on impact of *TERT* gene- smoking interaction on GC risk. Previously, some studies have investigated the interaction between other genes and smoking on GC, including tumor necrosis factor (*TNF*) gene [24], cytochrome P4501A1 (*CYP1A1*) [25], Interleukin-10 (*IL- 10*) [26], excision repair cross-complementing group 8 (*ERCC8*) [27] and Glutathione S-transferase P1 (*GSTP1*) [28]. These studies all suggested that gene polymorphism may play an independent role in gastric carcinogenesis and this genetic effect is exacerbated by cigarette smoking, which was similar with the results obtained in our study. Loci that are located nearby on the same chromosome may be in LD, which means that alleles at these loci are not inherited in an independent manner but certain allele combinations occur more often than expected by random segregation. The implication of LD in association studies is that knowledge of variation at a certain position also gives knowledge of variation at linked loci. In this study we also conducted the haplotype analysis for the rs2736100 and rs2736109, and we found that a haplotype containing the rs2736100- G and rs2736109- A alleles were also associated with a statistically increased GC risk.

Our study has several limitations. Firstly, the results obtained in this study should be verified with more subjects and in additional populations. Secondly, more SNPs within *TERT* gene should have been included in the analysis. Thirdly, other environmental factors should be included in the gene- environment interaction detections in the future studies.

In conclusion, our findings suggested that G allele of rs2736100 and G allele of rs2853669 in *TERT* gene, interaction between rs2736100 andsmoking and haplotype containing the rs2736100- G and rs2736109- A alleles were all associated with increased GC risk.

## MATERIALS AND METHODS

### Subjects

All study participants were recruited between June 2010 and March 2014 from the Affiliated Hospital of Qingdao University. Patients who had secondary or recurrent tumors, tumors of an unknown origin, history of other tumors or any histopathological diagnosis other than GC were excluded. Controls were randomly selected from healthy individuals from community-based chronic non-communicable diseases screening program conducted with nearly 1:2 matched (age and sex) in the same city. Consequently, a total of 360 diagnosed GC cases and 728 age and sex frequency-matched controls were included in current study. Mean age of all participants was 61.4 ± 14.5 years. Questionnaire investigation was conducted for all participants, and data on demographic information, clinical and biochemical index for all participants were obtained. Body weight and height were measured. Body mass index (BMI) was calculated as weight in kilograms divided by the square of the height in meters. Cigarette smokers were those who self-reported smoking cigarettes at least once a day for 1 year or more. Alcohol consumption was expressed as the sum of milliliters of alcohol per week from wine, beer, and spirits. Blood samples were collected from each participant in the morning after at least 8 hours of fasting. Informed consent was obtained from all participants.

### Genomic DNA extraction and genotyping

The SNPs were selected based on the NCBI database (http://www.ncbi.nlm.nih.gov/projects/SNP) according to the following two criteria: 1) located in a gene fragment that could have functional effects, and 2) with a minor allele frequency (MAF) of at least 5% in Chinese population, 3) previously reported associations with any type of cancer. Genomic DNA from participants was extracted from EDTA-treated whole blood, using the DNA Blood Mini Kit (Qiagen, Hilden, Germany) according to the manufacturer's instructions and stored at -20°C until use. The genotyping for four TERT SNPs (rs2736100, rs2853669, rs2735940 and rs2736109) were detected by polymerase chain reaction-restriction fragment length polymorphism (PCR–RFLP) method. The nucleotide sequence of primers and description for the 4 SNPs were shown in Table 7. The reaction volume was 25 mL, including 125 ng gDNA, with 0.5 μM of both primers, 0.2 mM of each dNTP, 1X PCR buffer, 1.5 mM MgCl_2_, and 0.5 unit (U) Taq DNA polymerase (Shanghai Keith Tell biological science and Technology Co., Ltd.). The following PCR cycling conditions were used: an initial denaturation step of 5min at 95°C; 30 cycles of 30 s at 94°C, 30 s at 57°C (for rs2736109 and rs2735940) or 56°C (for rs2736100 and rs2853669), and 30 s at 72°C; a final elongation step of 7min at 72°C. As a negative control, PCR mixture without gDNA sample was used to provide a contamination free PCR product. After approval of successful PCR amplification by 1.5% agarose gel electrophoresis, each PCR product was digested overnight with 10 U of restriction endonuclease enzymes (Shanghai Keith Tell biological science and Technology Co., Ltd.) at 37°C and electrophoresed on 3% agarose gel containing 0.5 μg /mL ethidium bromide and visualized under UV illumination. Genotyping results were confirmed by randomly assaying 5 % of the original specimens for replication to exclude genotyping errors. There were no discrepancies between genotypes determined in duplicate.

**Table 7:** Description and Probe sequence for 4 SNPs used for Taqman fluorescence probe analysis

SNP ID	Chromosome	FunctionalConsequence	Nucleotide substitution	Restriction enzyme	Primer sequences
rs2736100	5:1286401	Intron variant, upstream variant 2KB, utr variant 5 prime	T>G	SfcI	F:5′CCC CAC AAG CTA AGC ATT AT-3′R: 5′GAA GAA CCA CGC AAA GGA C-3′
rs2736109	5:1296644	Upstream variant 2KB	G > A	MboI	F:5′AAC ATC TGG GTC TGA GGT AGG 3′R: 5′TTA GGA TTA CAG GTC GCT CTT C 3′
rs2853669	5:1295234	Upstream variant 2KB	A > G	SacI	F: 5´- CAGCGCTGCCTGAAACTC-3R: 5´-GTCCTGCCCCTTCACCTT-´3
rs2735940	5:1296371	Upstream variant 2KB	T>C	MspI	F:5′ATC TTC TGC TTC CAT TTC TTC TC 3′R: 5′ TCG TCT TGT AAA TAC TTA GGA TTC C 3′

### Statistical analysis

All statistical analyses were performed by using the SPSS 16.0 software package (SPSS Inc, Chicago) for Windows (Microsoft Corp, Redmond, Wash). The means and standard deviation (SD) were calculated for normally distributed continuous variables and compared by Student's t test, and percentages were calculated for categorical variables and were analyzed using χ^2^ test. The χ^2^ test was also used to assess the deviation from Hardy-Weinberg equilibrium for genotype frequencies and to examine genotype and allele frequencies between cases and controls. Pairwise linkage disequilibrium (LD) analysis was conducted using SNPstats (http://bioinfo.iconcologia.net/SNPstats). Generalized multifactor dimensionality reduction (GMDR) was used to screen interaction combination among 4 SNPs within TERT gene and smoking. Logistic regression was performed to investigate associations between 4 SNPs within the TERT gene and to test for additional gene-environment interactions between rs2736100 and smoking, for GC risk. All reported *p*-values were two-tailed, and those less than 0.05 were considered statistically significant. To correct for multiple testing we defined a Bonferroni threshold: *p* = 0.0125.
